# Why the Gold Standard Approach by Mammography Demands Extension by Multiomics? Application of Liquid Biopsy miRNA Profiles to Breast Cancer Disease Management

**DOI:** 10.3390/ijms20122878

**Published:** 2019-06-13

**Authors:** Pavol Zubor, Peter Kubatka, Karol Kajo, Zuzana Dankova, Hubert Polacek, Tibor Bielik, Erik Kudela, Marek Samec, Alena Liskova, Dominika Vlcakova, Tatiana Kulkovska, Igor Stastny, Veronika Holubekova, Jan Bujnak, Zuzana Laucekova, Dietrich Büsselberg, Mariusz Adamek, Walther Kuhn, Jan Danko, Olga Golubnitschaja

**Affiliations:** 1 Department of Obstetrics and Gynaecology, Jessenius Faculty of Medicine, Comenius University in Bratislava, Martin University Hospital, 03659 Martin, Slovak Republic; tbielik57@gmail.com (T.B.); Erik.Kudela@jfmed.uniba.sk (E.K.); marek.samec@gmail.com (M.S.); alenka.liskova@gmail.com (A.L.); Dominika.Vlcak@gmail.com (D.V.); tatiana.kulkovska@gmail.com (T.K.); igor.stastny@uniba.sk (I.S.); zuzana_laucekova@yahoo.com (Z.L.); danko@jfmed.uniba.sk (J.D.); 2 Division of Oncology, Biomedical Center Martin, Jessenius Faculty of Medicine, Comenius University in Bratislava, 03601 Martin, Slovak Republic; zuzana.dankova@jfmed.uniba.sk (Z.D.); holubekova@jfmed.uniba.sk (V.H.); 3 Department of Medical Biology, Jessenius Faculty of Medicine, Comenius University in Bratislava, 03601 Martin, Slovak Republic; 4 Department of Pathology, St. Elizabeth Cancer Institute Hospital, 81250 Bratislava, Slovak Republic; kkajo@ousa.sk; 5 Biomedical Research Centre, Slovak Academy of Sciences, 81439 Bratislava, Slovak Republic; 6 Center for Cancer Prevention, 03659 Martin, Slovak Republic; polacek@jfmed.uniba.sk; 7 Department of Radiology, Jessenius Faculty of Medicine, Comenius University in Bratislava, 03659 Martin, Slovak Republic; 8 Department of Obstetrics and Gynaecology, Kukuras Michalovce Hospital, 07101 Michalovce, Slovak Republic; janbujnak@hotmail.com; 9 Oncogynecology Unit, Penta Hospitals International, Svet Zdravia, Michalovce 07101, Slovak Republic; 10 Weill Cornell Medicine in Qatar, Qatar Foundation-Education City, Doha 24144, Qatar; dib2015@qatar-med.cornell.edu; 11 Department of Thoracic Surgery, Faculty of Medicine and Dentistry, Medical University of Silesia, 40055 Katowice, Poland; m.adamek@e.pl; 12 Centre of Obstetrics, Gynaecology and Gynaecologic Oncology, DonauIsar Klinikum Deggendorf-Dingolfing-Landau, 94469 Deggendorf, Germany; walther.kuhn@donau-isar-klinikum.de; 13 Radiological Hospital, Rheinische Friedrich-Wilhelms-University of Bonn, 53105 Bonn, Germany; Olga.Golubnitschaja@ukbonn.de; 14 Breast Cancer Research Centre, Rheinische Friedrich-Wilhelms-University of Bonn, 53105 Bonn, Germany; 15 Centre for Integrated Oncology, Cologne-Bonn, Rheinische Friedrich-Wilhelms-University of Bonn, 53105 Bonn, Germany

**Keywords:** breast cancer, screening, liquid biopsy, omics, multi-level diagnostics, individualized patient profile, miRNA, mammography, predictive and preventive approach, personalized medicine

## Abstract

In the global context, the epidemic of breast cancer (BC) is evident for the early 21st century. Evidence shows that national mammography screening programs have sufficiently reduced BC related mortality. Therefore, the great utility of the mammography-based screening is not an issue. However, both false positive and false negative BC diagnosis, excessive biopsies, and irradiation linked to mammography application, as well as sub-optimal mammography-based screening, such as in the case of high-dense breast tissue in young females, altogether increase awareness among the experts regarding the limitations of mammography-based screening. Severe concerns regarding the mammography as the “golden standard” approach demanding complementary tools to cover the evident deficits led the authors to present innovative strategies, which would sufficiently improve the quality of the BC management and services to the patient. Contextually, this article provides insights into mammography deficits and current clinical data demonstrating the great potential of non-invasive diagnostic tools utilizing circulating miRNA profiles as an adjunct to conventional mammography for the population screening and personalization of BC management.

## 1. Introduction

**Figure 1:**
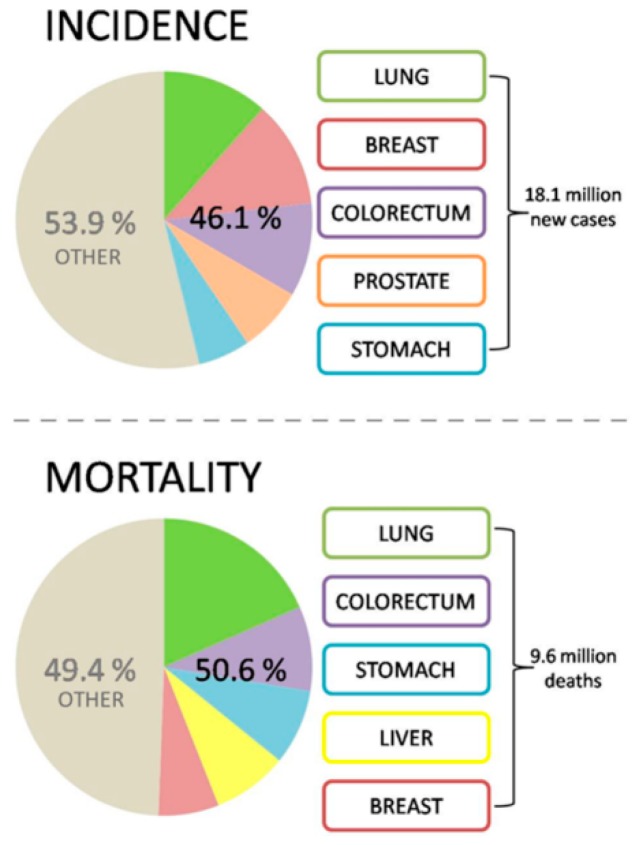
Global cancer statistics demonstrating the most prevalent cancer types; the image is based on the data published by 2019 [1].

Cancer is one of the leading healthcare burdens worldwide. In 2018 18.1 million (95% UI: 17.5–18.7 million) new cases of cancer (17 million excluding non-melanoma skin cancer) and 9.6 million (95% UI: 9.3–9.8 million) cancer related deaths (9.5 million excluding non-melanoma skin cancer) have been estimated worldwide. Figure 1 summarizes most frequent cancer types [1]. To this end, a big portion of cancer-related deaths can be avoided at the level of primary prevention: innovative screening programs and targeted preventive measures are essential tools to identify and mitigate modifiable risks individually and in a timely manner [2,3,4].

Further, at the level of secondary prevention, cancer-diagnosed patients could demonstrate longer survival rates and have much better quality of life if the disease-management process would adapt treatment algorithms that are tailored exactly to the individualized patient profiles [5,6]. For the population screening, early and predictive diagnosis, as well as prognosis and disease monitoring, a multi-level diagnostics (multi-omics, sub-cellular and medical imaging) method utilizing the great information potential of liquid biopsy is considered to be the most appropriate tool [7] and is thoroughly analyzed in the current paper using the example of breast cancer (BC) management.

## 2. Breast Cancer in the Context of Global Cancer Mortality

The 28 Member States of the European Union (EU-28) with a population of 504.6 million had 5.0 million deaths in 2012 with more than one fourth attributable to cancer [8,9]. Detailed analyses revealed 29.2% of deaths among men and 22.5% of deaths among women were caused by cancer alone in 2012. Next to the disorders of the circulatory system (cerebrovascular and heart diseases), cancer is the second most common cause of deaths in the EU, being one of the major public health burdens in the European Union (EU). According to the International Agency for Research on Cancer (IARC, 2012), out of 1.26 million cancer-related deaths in the EU-28, breast cancer (BC) alone is responsible for 91,500 deaths annually [10]. In the global context, the epidemic of BC is associated with a number of external and internal risk factors attributed to the early 21st century [11].

BC is the most frequent tumor in female populations worldwide, with an incidence rate of 43.1 per 100,000 world age-standardized rate (ASR-W), a mortality rate of 12.9 per 100,000 ASR-W, and a 5-year prevalence of 239.9. In low-, middle- and high-income countries the incidence rates are persistently increasing [12]. To this end, the contribution to the BC incidence by the European Region is higher than the global average [13]. Specifically for the EU-28, the incidence and mortality rates are as high as 80.3 and 14.4 per 100,000 ASR-W, respectively [14]. Most of EU-28, including the biggest sufferers such as the UK [15], France [16], Italy [17], Germany [18], and Belgium [19], have established national programs for BC screening by mammography as the golden standard for reducing mortality from BC. Evidence shows that national mammography screening programs have sufficiently reduced BC related mortality [20,21]. Therefore, the adequacy and usefulness of the mammography-based screening for women aged by 50 to 74 years is generally well-accepted [22]. Therefore, the great utility of the mammography-based screening is not an issue. However, it is important to decide for which population the mammography-based screening should be considered to be optimal i.e., the “golden standard” approach (see Table 1). Both false positive and false negative BC diagnosis [23], excessive biopsies and irradiation linked to mammography application, as well as sub-optimal mammography-based screening, e.g., in case of high-dense breast tissue in young females [24], altogether increases awareness among the experts regarding the mammography-based screening limitations. Severe concerns regarding the mammography as the “golden standard” approach demanding complementary tools to cover the evident deficits [25] led us to present innovative strategies, which would sufficiently improve the quality of the BC management and services to the patient. 

Contextually, this article is aiming to provide insights into mammography deficits and current clinical data demonstrating the great potential of non-invasive diagnostic tool utilizing circulating miRNA profiles as an adjunct to conventional mammography for the population screening and personalization of BC management [6]. To this end, innovative screening strategies should consider primary [11] and secondary [26] levels of predictive and preventive medical approach, including both non-modifiable and modifiable risk factors [27] based on comprehensive individual patient profiles that means application of multi-omics [28,29], big data processing [30] and artificial intelligence such as machine learning approach [5]. Table 1 summarizes the categories of women with the mammography screening applicability.

## 3. Breast Cancer Screening by Mammography: An Evolution

Radiography is the oldest and most common form of medical imaging. A mammography is an x-ray medical method examining the breast using lower doses of radiation. Because these x-rays do not pass through breast tissue easily, the mammogram machine has two plates that compress the breast to spread the tissue apart, resulting in a more accurate image with less radiation [31]. Over the past decades, technology changed over from the analog to digital picturing. 

The analog mammography used film as both a receptor and a display for the image to produce static, fixed images. The advantage of analog mammography linked with computed radiographic systems included much less costs than digital mammography, however the main disadvantage, that the image is far inferior to digital mammography and also the storage in protective sleeve required large amounts of space, was the reason for leaving it worldwide. Thus, the digital technology fluently re-placed the analog mammograms. 

**Figure 2:**
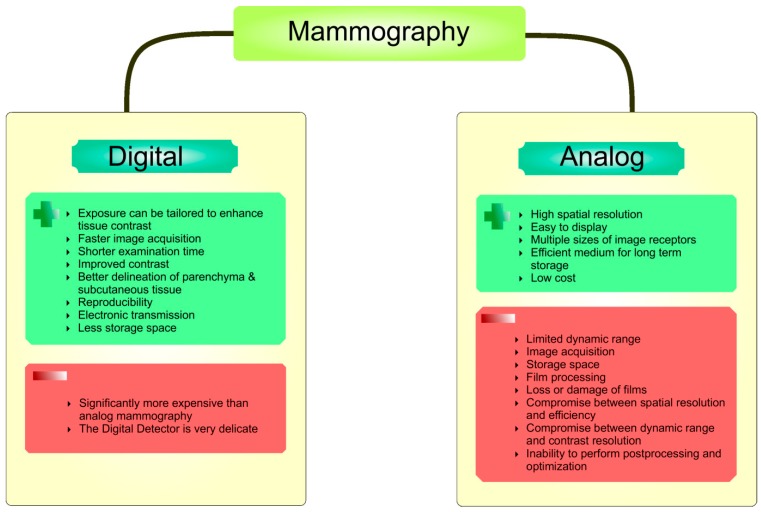
Advantages and disadvantages of digital versus analog mammography.

Digital mammography uses detectors that change x-rays into electrical signals (pixels), which are transferred into a digital receptor and converts x-rays energy into numbers. It produces an image displayed on a monitor or printed on high-resolution printer. In comparison with analogue film, digital mammography provides images with more contrast, allows image manipulation, and archive films which reduces the risk of misplacement or damage. Moreover, the digital detector provides a crisp image with no limitations on breast size and detects cancer cells earlier than analog mammography. This makes this method superior to film mammography [32,33]. Digital breast tomosynthesis mammography (DBT) is relatively new technology being developed to improve detection and characterization of breast lesions, especially in women with non-fatty breasts. The x-ray dose for a tomosynthesis image is similar to that of a regular mammogram. DBT creates a 3-dimensional (3D) picture of the breast using several low dose x-rays obtained at different angles. The breast is positioned and compressed in the same way as in a regular mammography, but unlike for regular mammography, the x-ray tube moves in a circular arc around the breast in DBT [34,35]. DBT provides an advantage in detection of breast masses compared to 2-dimensional (2D) mammography, since it allows the separation of the tissue layers and the noticeable reduction of occlusions caused by overlapping anatomical structures. While DBT slice images provide advantages for detecting mass lesions, it is more difficult to get an overview and evaluate the distribution of microcalcifications compared to 2D mammography images. Therefore, the parallel using of 2D mammography and DBT slice images seems to be necessary in clinical diagnostic practice. Due to the cumulative patient dose, using both 2D mammography and DBT at one session is not an acceptable screening method. However, the DBT method offers a possibility of reprocessing the tomosynthesis data to create a 2D mammography-like image (synthetic mammography, SMMG) from DBT image data at the dose level of a single DBT screening. The use of SMMG with DBT provides significant benefit of increased diagnostic accuracy compared with regular mammography [36]. Here, the initial evidence suggests that SMMG may reduce recall rates and increase cancer detection rates when added to digital MMG screening [37]. Figure 2 describes the advantages and disadvantages of analog versus digital mammography.

Regular screening for BC with mammography (along with other examinations such as breast self-examinations) is widely recommended with the aim to reduce mortality of BC. Although controversy remains over the best screening programs and to whom it should be offered (e.g., for the primary cancer free population, screening of secondary cancer after previous BC surgery, screening of population with different genetic background or different age-groups of screened population), these methods are regularly used in clinical practice, following local or national guidelines. Reflecting this, there are multiple approaches for BC screening. E.g., according to the Canadian Task Force on Preventive Health Care, the use of mammography for women at average risk of BC aged 40–74 years includes following recommendations: for women aged 40–49 years, it is recommended to not routinely screen with mammography; for women aged 50–69 years and also 70–74 years, it is recommended to routinely screen with mammography every two to three years [38].

Although the results of mammographic screening in women aged 50–70 years are sometimes disputed [39], there is a consensus among clinicians that BC screening of women in this age group is effective. On the other hand, there is no consensus about the value of BC mammographic screening among women who are aged 40–49 years having denser breasts compared to postmenopausal women [40]. Regarding women under 40 years of age, in which BC is rare and typically presents symptomatically, the best imaging modality is controversial. Routine screening of women in this age group in the absence of significant BC risk factors is not recommended. Some authors showed the superior sensitivity of ultrasound screening for BC in women under the age of 40 years; however, they noted that mammography and/or MRI remain essential adjuncts, particularly in the identification of multifocal disease [41]. The large body of analysis comparing ultrasound and mammography to evaluate women aged 30–39 with symptoms of possible BC has demonstrated that ultrasound screening is a superior diagnostic tool [42], which may impact on the consideration of current clinical practice guidelines that nowadays recommend mammography as the first evaluation in these women.

A special group for screening is women under 50 years who underwent breast conservation therapy, as those women may benefit from breast screening as an adjunct method to MMG. In this regard, the addition of MRI to annual MMG screening improves the detection of early-stage but biologically aggressive BC [43].

The last group, where much of the variability in BC screening programs exists, represents the high-risk population. The annual mammography in women with one or two first degree relatives with invasive BC starts 5 to 10 years younger than the youngest case in the family, but no earlier than age 25 and no later than age 40. Women with a breast biopsy showing atypical hyperplasia or lobular carcinoma in situ and following surgical management to rule out invasive carcinoma have annual mammography. Women with a history of chest wall radiation (i.e., mantle radiation for treatment of Hodgkin’s lymphoma) at age 30 or younger have an annual mammography and breast screening MRI starting 5 to 10 years after radiation given, but starting no earlier than age 25 and no later than age 40. For women with *BRCA1* or *BRCA2* genes mutations, current guidelines recommend annual screening by clinical breast examination and mammography starting at age 30 [44]. However, these approaches may vary from country to country.

## 4. Breast Cancer Screening by Mammography and Profiling of Genetic Risk

Routine BC screening is recommended for women from the age of 50 years; however, high-risk individuals (with a strong family history of the disease) may be included for screening at earlier age. About twenty percent of all BCs occur in women under 50 years old, and the vast majority of these women do not have any family history of the disease. Most of these tumors have poorer prognosis, therefore early diagnosis by mammography screening, irrespective of known family history, can be clinically beneficial due to reduced BC mortality [45]. Covering the genetic risk assessment into mammography screening programs (by modification of screening frequency or using alternative modalities such as MRI and USG) has been supposed as the way which maximize benefits and minimize harms [46]. Therefore risk-stratified mammography screening based on genetic risk seems to be more effective compared to prevailing age-stratified approaches [47].

### 4.1. Low- and Intermediate-Risk Women

Intermediate-risk women include cases with a breast biopsy that shows changes such as atypical ductal or lobular hyperplasia, or lobular carcinoma in situ. A calculated risk of BC in these women is ranged from 20% to 29% based upon family history, personal health history, or certain genetic markers. Average-risk women (low-risk women) with none of the above risk factors have a 10–13% lifetime risk of BC [48]. Mammography screening in low-or intermediate-risk women aged less than 50 years is intensively discussed. Arguments for the lower age of mammographic screening include the individual and societal gains linked with increased survival rates, greater work life participation, and lower treatment costs due to early detection. On the other hand, arguments against the lower age of mammography screening include the possible harms and higher costs of full population screening. In this regard, screening in this specific age group of women is accompanied by more cases of false-positive results and unnecessary biopsies because of lower screening specificity. A recent review by Nelson et al. [49] assessed the studies of screening in intermediate-risk women, including mammography screening. Results demonstrated that false-positive results are common and are higher for annual screening, younger women, and women with dense breasts. It seems that the absolute benefits (e.g., number of deaths prevented) are smaller than for older women, because of general lower BC incidence and lower sensitivity of mammography in women aged 40–49 years [33,50].

Several older clinical studies did not demonstrate a significant reductions in BC mortality resulting from screening low-risk women aged 40–50 years [51,52]. A more recent study of Moss et al. [53] enrolled women aged 39–41 years from 23 UK NHS Breast Screening Programme units. Participants were randomly assigned to intervention groups with annual screening by mammography up to an age of 48 years, or to a control group receiving usual medical care (invitation for screening at age 50 years and every 3 years thereafter) respectively. Results showed a significant reduction in BC mortality in the intervention group compared with the control group in the first 10 years after diagnosis but not thereafter from tumors diagnosed during the intervention phase. The overall BC incidence during 17-year follow-up was similar between the groups. A meta-analysis of eight trials revealed that mammography screening reduces BC mortality by 15% for intermediate-risk women aged 39 to 49 years [54].

### 4.2. High-Risk Women

Validated risk assessment models demonstrated that high-risk women are considered to be those with lifetime risk of BC that is greater than 20% and very high-risk women with a 30% or greater risk for the disease [55]. High-risk individuals include women with a known *BRCA1* or *BRCA2* mutation and their first-degree relatives, women with a personal history of invasive BC or ductal carcinoma in situ and lobular carcinoma in situ or atypical hyperplasia, Li-Fraumeni, Cowden/*PTEN* or Bannayan-Riley-Ruvalcaba syndrome (and first-degree relatives), mutation in specific genes (*ATM*, *CDH1*, *CHEK2*, *NBN*, *NF1*, *PALB2*, *PTEN*, *STK11* or *TP53*), and a history of chest irradiation between the age of 10 and 30 [56]. A germline gene mutation in *BRCA1* or *BRCA2* results in a significantly elevated lifetime risk of developing breast and ovarian cancer estimated at up to 7 and 25 times, respectively, compared to average risk population. It is supposed that more than 90% of hereditary cases of BC (and also ovarian cancer) are a result of a mutation in *BRCA1/2* [57]. The estimated prevalence of *BRCA1* and *BRCA2* mutations is dependent on the population and can vary between 1 in 300 and 1 in 800, respectively [58]. 

Meta-analysis of the three studies that compared MRI plus mammography versus mammography alone in screening of young women at high BC risk revealed the sensitivity of MRI plus mammography to be 94% (95%CI 86–98%) and the incremental sensitivity of MRI to be 58% (95% CI 47–70%) [59]. Regarding the high-risk healthy women over the age of 50, there are no clear-cut guidelines for how to continue screening them. Most clinicians continue to screen these women with annual MRI, moreover, in some older women, mammary gland tissue becomes less dense, making it easier to recognize lesions using mammography [60].

Management of high-risk women for the development of BC is debatable, mainly in women carrying a *BRCA1/BRCA2* or *p53* genes mutation because they can develop cancer at an earlier age [61]. This is because mammography alone has limitations in screening younger women with a specifically denser mammary gland tissue or with special tumor phenotypes [60]. Therefore, magnetic resonance imaging can be used along with mammography in these women to increase sensitivity of the screening program. Regarding the high-risk individuals with *BRCA1* or *BRCA2* mutations, current guidelines suggest to begin annual MRI imaging at age 25 and to add mammography at age 30 [44]. Recent meta-analysis of Phi et al. [62] showed that additional screening sensitivity from mammography above that from MRI is limited in *BRCA1* mutation carriers. On the other hand, mammography contributes to screening sensitivity in *BRCA2* mutation carriers, especially those over 40 years. Authors summarized that a differential screening schedule by BRCA status is worth considering [62]. The results of a prospective multicenter trial enrolling 296 carriers of the *BRCA1/2* mutation showed that carriers of the *BRCA* mutation younger than 40 years may not benefit from full-field digital mammography surveillance in addition to dynamic contrast agent-enhanced MR imaging [63].

Based on above-mentioned clinical data, we can conclude than women at high-risk of BC require a close breast surveillance. On the other hand, there is no evidence that more frequent mammography screening or screening with other modalities actually reduces the risk of BC mortality in women with an intermediate or low BC risk (including women with extremely dense breast at mammography) [64]. In addition, mammographic screening has several weaknesses: (a) the risk of false positives; (b) the risk of false negatives; (c) X-ray radiation exposition may trigger BC in high-risk women; (d) mammography performance is operator dependent [65,66,67].

Moreover, clinical practice demonstrates common diagnostic problems in the distinguishing the pure atypical ductal hyperplasia from advanced lesions, such as DCIS and/or invasive ductal carcinoma following a mammography, and even combined with follow-up core needle biopsy. In this regard, accumulating evidence from oncological research confirming the role of miRNAs in BC progression should be helpful. Therefore, it seems logical to use the potential of miRNA molecules as biomarkers in early BC detection as a follow up of mammography and core needle biopsy [68]. 

For women who are at high risk for BC and are unable to undergo an MRI evaluation (or are pregnant), ultrasonography of the breast is considered as a useful diagnostic tool. Moreover, ultrasound has been suggested as an adjunct screening method that can detect BC that is missed when using mammography. In this regard, Health Quality Ontario [69] investigated the benefits of ultrasound as an adjunct to mammography compared with mammography alone in women at average and high BC risk. After including five prospective studies, authors concluded that there is low-quality evidence that screening with mammography and adjunct ultrasound detects additional cases of disease, with improved sensitivity compared to mammography alone. Moreover, the results did not show that the use of ultrasound as an adjunct to mammography might reduce BC-related mortality in high-risk women. Due to certain limitations of mammography, particularly in women with dense breasts, ultrasound in combination with contrast-enhanced magnetic resonance imaging, are suggested to supplement mammography for the early detection of BC [70].

### 4.3. Genetic Profiling as a Tool for the Risk Assessment

Another method focused on the more specific and early detection of BC itself and its risk assessment involves genetic signature profiling. It includes either only genetic variants, or gynecological characteristics or it combines all these factors together. The first mentioned genetic model is mainly the BRCAPRO with several modifications [71] and BOADICEA [72]. Both are able to predict the risk of the disease by mutations analyses in highly penetrant genes, such as *BRCA1* and *BRCA2*. These variants have high individual benefit, but due to rare incidence, they are not suitable for general screening. Therefore, other genetic models include several single nucleotide polymorphisms (SNPs) in low penetrant genes, as they are more frequent in the general population and thus are preferable in the primary screening programs. Some models analyze 7 [73], 12 [74], 51 [72], 77 [75], 88 [76] or 153 SNPs [77]. However, the predictive ability of these genetic models, explained by an area under the ROC curve (AUC) is individually low, ranging from 0.53 to 0.68 [75,77]. When combined multiplicatively with other risk models (i.e., BRCAT / Gail model), a substantial improvement in specificity and sensitivity was observed [72,73,76,78]. Addition of a genetic risk model (12 SNPs) to the BRCAT had a greater effect among African Americans than in whites as it reclassifies the high-risk status of several women undergoing screening mammography [74]. 

Comparative analyses focusing on the evaluation of the best model’s discriminative ability explained by the area under the ROC curve, including genetic, Gail/demographic, and mammography models, revealed the domination of single mammography model above other models. Better identification of women with elevated risk for BC could be attained by the combination of these models as it increases the AUC values by a statistically significant amount [79,80,81]. It seems that BC risk-stratification based on the combination of mammography screening, genomics and classical risk factors could augment comprehensive risk prediction, provide many benefits in further treatment or monitoring and facilitate tailored preventive intervention.

### 4.4. Proteomic Profiling as a Tool for Screening Guidelines

The clinical complications due to increased breast density lead to confusion for physicians in the management of women with dense breasts and their follow-up. In this regard, the discussion between patients and health-care providers regarding the need for supplemental screening is necessary. A biochemical clinical approach not affected by density of mammary gland or risk profile of the women would provide an important tool in the management of women with dense breasts or other risks and doubtful imaging results. With the discovery of key biomarkers and protein signatures for BC, proteomic technologies are fully available to provide an ideal diagnostic adjunct to imaging. Research studies have demonstrated that breast tumors are linked with complex changes in the levels of both serum protein biomarkers (SPB) and tumor associated autoantibodies (TAAb) [82]. 

Recently, Videssa^®^ Breast as a combinatorial proteomic biomarker assay has been comprised of SPB and TAAb integrated with patient-specific clinical data to produce a diagnostic score that reliably detects BC as an adjunctive tool to imaging. Certain blood-based biomarkers are associated with higher mammographic density [83], therefore it is unknown whether the biomarkers included in Videssa^®^ Breast might be impacted as well. Reese et al. [84] aimed to assess the performance of Videssa^®^ Breast in women with dense and non-dense breasts and determine whether this test could help as an additional tool to clinicians in managing women with dense breasts and questionable imaging results. Results of this study demonstrated that Videssa^®^ Breast has high sensitivity and specificity in detecting BC, irrespective of density status. Moreover, a negative Videssa^®^ Breast test gives an assurance to women with dense breasts that they likely do not have BC. 

In addition to above mentioned study, Lourenco et al. [85] conducted two prospective clinical trials with the aim to assess a blood-based Videssa Breast test for accurately detection of BC and reduce false positives imaging results. Moreover, they used the Videsa test to detect BC for use in conjunction with imaging to aid healthcare providers in making informed decisions on treating young women (under 50 years old) with difficult-to-assess imaging findings. Authors showed that Videssa Breast can effectively detect BC when used in combination with imaging, improves the management of BC in individuals under 50 years old with challenging or absent imaging findings, and can apparently decrease undesirable clinical procedures. The aforementioned results pointed to the benefit of the integration of SPB and TAAb data in BC diagnosis. Moreover, these data sustain the further progress of combinatorial proteomic approaches for detecting BC.

## 5. Liquid Biopsy as Marker for Breast Cancer Control and Management

Traditional BC diagnostic tools include clinical and physical examinations, imaging mammography, ultrasound, and/or magnetic resonance imaging, followed by histopathology. Ultrasound as a non-invasive and safe tool is very helpful, but since it is unable to screen the general population for cancer, it cannot replace mammograms, especially in women above 40. Nevertheless, once a suspect lesion in the breast is diagnosed, the bioptic verification is necessary. 

Histopathology as an invasive approach to examining cancerous tissues once the disease is installed has for decades been a golden standard for assessment of the tumor biology and if available can also facilitate assessment of ipsilateral lymph node status and serve as a decision-making tool in disease management. Currently, the histological and partial genetic profile of solid tumors is achieved from biopsy or surgical excisional specimens, but these invasive techniques cannot always be performed routinely. It is well-known that tumors consist of subpopulations of cells and needle biopsy takes only a small amount of tumor tissue that does not reflect its full heterogeneity, making the capturing of aggressive clones problematic. Moreover, neither tumor cells show heterogeneity, nor their metastases, which carries different genomic aberrations. As core biopsy of tumor tissue reveals only the portion of this heterogeneity, especially in patients with metastases and in overall assessment it does not seems to be fully representative [86,87], except of full excisional biopsy.

Knowledge, that cancer tissue is associated with mutations in genes, specific genetic alterations and protein expressions, together with those needle biopsies in BC diagnostics have some disadvantages leading to false decisions, sets the identification of other tumor biology markers as useful tools for diagnostic, prognostic and therapeutic purposes. In line with this, other information indicates that surgically resected primary tumor alone does not provide sufficient information about the future diseases biology and seeding of metastases, which can be dissociated at different sites and can harbor unique genomic characteristics that are not detectable in the corresponding primary tumor of the same patient. Thus, the international oncology community is in active pursuit of non-invasive methods for the diagnosis and monitoring of BC patients, which could be introduced in clinical practice. Nowadays efforts are focusing on monitoring specific bodily fluid biomarkers for early and minimally invasive detection [88]. The background for this is the natural behavior of the malign disease, leading to its spread. As disease advances, tumor cells are released from primary tumors (e.g., circulating tumor cells – CTC) and/or metastases or tumor cells release their own nucleic acids (DNA, RNA, miRNA, etc.) into the circulation (circulating free DNA – cfDNA). Analysis of these particles with tumor origin has led to a new diagnostic procedure known as the Liquid Biopsy [89,90].

Early detection of BC disease, treatment of BC, and metastasis monitoring are of eminent importance to ensure favorable prognosis in an individual. Although conventional diagnostic methods, i.e., breast X-ray mammography is precise (“gold standard”) clinical method, they may bring about radioactive/invasive harms in patients. In this regard, liquid biopsy as a noninvasive approach is convenient for repeated sampling in clinical oncology practice. Emerging interests in “liquid biopsies” have encouraged researchers to recognize and develop clinically-valid noninvasive genomic and epigenomic signatures that can be exploited as biomarkers capable of detecting premalignant and early-stage tumors, or as biomarkers for prognostic and metastatic evaluation, including cancer relapse monitoring [91]. Importantly, these genomic and epigenomic signatures that are frequently deregulated in cancers, have great potential to serve as promising entities for multifarious purposes within clinical oncology [92].

The term “liquid biopsy” refers to the use of circulating (cell-free) tumor DNA (ctDNA), circulating tumor cells (CTCs), and other non-invasive biomarkers such as long non-coding RNAs (lncRNA), messenger and microRNAs (mRNAs and miRNAs), proteins (soluble or membrane-associated proteins and glycoproteins) and exosomes for the early diagnosis, prognosis, monitoring of clinical progression and response to treatment [93] as was demonstrated in various cancer types including HPV-Associated oropharyngeal cancer [94], and BC [95,96,97]. Thus, liquid biopsy can be used as an additional diagnostic tool to core or excisional biopsy of primary tumor or its metastasis, or indirect diagnostic tool in case of technically non-performable, non-achievable localization of tumor/metastasis. The high importance for wide clinical application of liquid biopsy supports the results from the studies analyzing temporal and spatial heterogeneity of the tumor tissue. Several studies have described the important role of CTCs in the clinical management of BC disease, notably the ones in association with primary metastases [98,99]. On the other hand, Mansouri et al. [100] evaluated whether CTCs may serve as a clinical prognostic marker for survival in primary BC. Their meta-analysis pointed to CTCs as valid prognostic marker in primary BC prior to any systemic therapy mainly when it is studied through CellSearch^®^ using, concluding that the more the CTCs are linked with increased death and relapse rates in patients. In another study, the prognostic value of CTCs with an epithelial-mesenchymal transition (EMT) phenotype (expression of *TWIST1*, *SNAIL1*, *SLUG*, *ZEB1* transcription factors was analyzed) in primary BC patients were assessed [101]. CTC EMT was determined in 77 from 427 (18.0%) patients. Considering all subgroups of patients, individuals without detectable CTC EMT in peripheral blood manifested longer disease-free survival compared to patients with detectable CTC EMT. Likewise, plasma DNA mutations in ER  +  MBC seems very promising markers in the early prediction of therapeutic response. In this regard, Kumar et al. [102] used digital PCR-based target enrichment, which was followed by next-generation sequencing to analyze plasma DNA mutations in *ESR1*, *PIK3CA*, and *TP53* in a prospective cohort of 58 patients with ER  +  MBC. This assay found *ESR1*, *PIK3CA*, and *TP53* plasma ctDNA mutations in 55%, 32%, and 32% of individuals and revealed ctDNA mutant allele fractions that were frequently discordant among the analyzed genes. 

**Figure 3:**
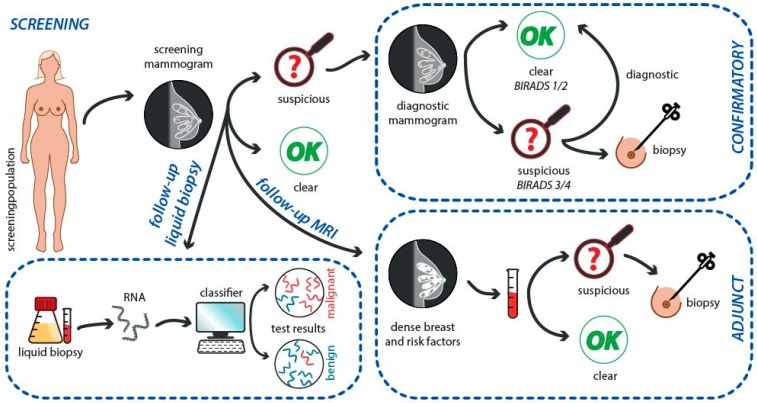
Liquid biopsy miRNA adjunct to conventional screening of BC within personalized diagnosis and improved management of the disease.

Despite the initial optimism and expectancy due to identification of CTCs and ctDNA from liquid biopsies in cancer patients, most recent data indicate that although these markers provide a high grade of cancer specificity, both groups of clinical indicators are rare in body fluids. Thus, these markers may be insufficient as clinically valid diagnostic markers. In general, ctDNA represents only less than 1% of the total cfDNA detected in body fluids. In this regard, the ratio of CTCs to white blood cells consists approximately 1:1 million [103]. Thus, a study that assessed the ability of ctDNA to recognize specific mutations in patients with primary tumors demonstrated positive result in only 73% of colorectal, 57% of gastroesophageal, and 48% of pancreatic carcinomas [104]. These data may be considered rather disappointing contemplating the fact that each of these mutations were known apriori before screening [105]. Importantly, other molecules derived from tumor mass, such as non-coding RNAs (including miRNA) that are far more plentiful than ctDNA or CTCs in body fluids, are relatively stable in biofluids. These RNA molecules are often deregulated, even in the initial stages of carcinogenesis. These features favor RNA markers (when compared to CTCs and cfDNA) for further methodical development as noninvasive liquid biopsy diagnostic and prognostic biomarkers for cancer disease, including BC. In addition, liquid biopsy miRNA biomarkers are applicable only for cancer patients but for also healthy individuals with benign diseases. Thus, cancer screening, staging, and response to treatment may be more effectively assessed by evaluating specific miRNA expression levels in body fluids [106]. The role of liquid biopsy analyzing miRNA signatures as adjunct to conventional screening of BC is summarized in Figure 3.

### Extracellular miRNA Molecules as an Important Tool of Liquid Biopsy in BC Screening

**Figure 4:**
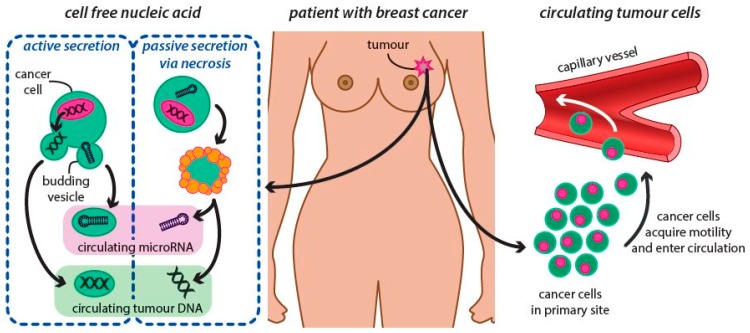
The background of the natural behavior of the malign BC disease leading to its spread through the circulating tumor cells and consequent secretion of cell free nucleic acids.

Circulatory tumor cells (CTCs) come either from primary or metastatic cancer tissue (Figure 4). In addition to previously studied ctDNA and CTCs there are also other circulating nucleic acids. The presence of circulating cell-free miRNA (cfmiRNA) molecules in plasma is the latest knowledge in the liquid biopsy era studied in BC patients. 

MicroRNAs (miRNAs) are short, non-coding RNAs of typically 22 nucleotides in length, which regulate gene expression at the post-transcriptional level and thus are responsible for proteome shaping [107,108], regulating post-transcriptional gene expression by binding to the 3′ untranslated regions of mRNA [109]. The sequence that is crucial for this binding is known as the ‘seed sequence’, situated mostly at positions 2–7 of the miRNA 5′-end [110]. MiRNAs are encoded by genomic DNA and are located mostly in intergenic regions (about 52%), intronic regions of genes (40%) and within exons (8%) [111,112]. They represent the human genome information, previously considered to be junk DNA, nowadays believed to be the hidden treasure regarding their potential relevance in diagnosis, prognosis, treatment and follow-up of cancer [113], including BC [6]. The miRNAs were initially discovered in 1993 [114]. A few years later, human studies were launched when its role in cancer was described [115,116]. Since then, an enormous number of studies of miRNA role in various disease’s ethiopathogenesis was conducted, describing their roles in or connection to them, including women’s cancers. Nowadays, the miRBase, a searchable database of published miRNAs contains more than 1800 human miRNAs sequences [117,118]. 

The first reports describing the existence of a miRNA signature characterizing human BC were published in 2005, suggesting the involvement of miRNAs in the pathogenesis of this human neoplasm [119,120]. The following years of research showed that miRNAs play a vital role in tumor initiation, progression, drug resistance and disease metastasis. Moreover, the tissue and cancer specificity of several miRNAs enabled us to generate miRNA fingerprints for several cancer types in women, reflecting their reproductive organs [110,121,122,123], and this specific miRNA expression profile can better classify tumors as compared with the mRNAs. MiRNAs thus have not only important diagnostic purposes, but also high prognostic value, while opening new possibilities in the cancer management, treatment stratification, and in the designing of personalized therapy [108,110,124], most of all in BC [6].

## 6. Circulating miRNA 

Apart from the tumor microenvironment, miRNAs can be found and isolated from various body fluids including serum, plasma, saliva, urine, breast milk, seminal fluid, cerebrospinal fluids and others [125,126,127]. The miRNA molecules found in the circulation are derived from tumor tissue cells, cells with short half-life as platelets, broken cells after tissue injury, apoptotic or necrotic cells and chronic inflammation [128,129]. These miRNAs, called circulating miRNAs or extracellular miRNAs (ECmiRNAs), are typically contained within exosomes and vesicles or protein bound complexes, which are shed from tumor cells into the circulation. Packaging complexes protect RNA from degradation, making them remarkably stable [130] and resistant to RNases, fluctuations in pH, long storage periods and to multiple freeze/thaw cycles [125,131]. The stability is most probably caused by the transport mechanisms as miRNAs are conjugated in complexes, providing them the protection. ECmiRNAs can be released and transported a) in the membrane-derived vesicles - either in microparticles (microvesicles) or in smaller exosomes b) in HDL or LDL lipoprotein complexes c) in AGO protein complex or d) wrapped in large apoptotic bodies [132,133,134]. Such exported extracellular miRNAs can be taken up by variety of recipient even distant cells, where they can alter target gene expression. ECmiRNAs thus represent cell-cell communication, which contributes in carcinogenesis to tumor progression, metastasis and therapy resistance [107,108,125,135,136]. The export of miRNAs most likely has selective patterns and is not only passive. Cells secrete specific miRNAs due to cellular signals or environmental cues and load them into specific vesicles. Moreover, some miRNAs seem to be expressed only to be exported, as they were not detected in the parent cell, only in the extracellular vesicles [132,137].

Due to the above mentioned characteristics of the ECmiRNAs and non-invasive method of acquisition, and despite it only being several years since its discovery, extracellular miRNAs represent excellent detectable biomarkers and seem to be very promising useful biomarkers in non-invasive monitoring and management of BC [135,138,139].

Considering this, to evaluate extracellular miRNAs as a biomarker in BC, the sensitive detection method is essential. This is possible with several methods, e.g., high-throughput sequencing, quantitative real-time PCR (qPCR), digital PCR (dPCR) or microarrays on chip. All these techniques are now fully available, differing only in some requirements. For example, in qPCR experiments it is necessary to find a stable extracellular miRNA reference in plasma or serum of BC patients. To achieve this, a few miRNAs as miR-10b, -16, -30a, -103, -148b, -191, -192 or RNU6 are usually used as endogenous reference markers [140,141].

## 7. miRNAs as Potential Blood-Based Biomarkers for Early Breast Cancer Detection

It was proved that miRNA has a crucial role in development of breast tumors and that miRNA expression is highly deregulated either in tumor tissue, metastatic tissue, or in plasma from BC patients. It causes a loss of control for many biological processes such as proliferation, differentiation, apoptosis, epithelial-mesenchymal transposition with cell migration, and miRNA can play a specific role as a regulator of metastasis in many levels of metastatic cascade as well. On the other side, except oncogenic activities, miRNA also exhibits oncosupressor characteristics by targeting miRNA coding oncoproteins [142]. Reflecting this knowledge, circulating miRNAs can serve as one of the most promising biomarkers in oncology for early diagnosis, prognosis and therapeutic response prediction [143]. Thus, the biological employ of miRNAs in BC management as liquid biopsy marker recently obtained major interest due its advantages compared to other markers, both in translational and clinical research. 

Malignant tumors can stay clinically asymptomatic for quite a long time, until they reach the size to be clinically detected or spread to the distant organs forming metastases. There has been a worldwide effort to identify BC in its early stages for decades. This involved self-examination for a palpable mass and mammography screening (MMG), enriched by ultrasound. The advance in primary screening, mainly thanks to modification of approaches (MMG + genetic risk subdivided populations), enabled us to detect BC quite quickly, however, it is still necessary to undergo core-cut biopsy or fine needle aspiration to set the diagnosis and biological profile of the cancer [144]. Nevertheless, as was shown recently, mammography could not be an early screening tool exclusively, and there exist other possibilities, overcoming its limited specificity and sensitivity, e.g., liquid biopsy technology [68]. Thus, an ideal approach should be a combination of MMG and some sensitive based “liquid biopsy” biomarker. 

The definition of biomarker states that it is “a biological molecule found in blood, other body fluids, or tissues that is a sign of a normal or abnormal process or of a condition or disease” [145]. Such a marker should be readily accessible, sensitive enough to detect all types of tumors, and specific enough to not give false positive results [109]. Based on their usage, we can classify the BC biomarker as including risk screening, prognostic, predictive and diagnostic and disease monitoring biomarkers. Thanks to developments in molecular biology, new circulating biomarkers (ctDNAs, mRNAs, cell surface receptors, transcription factors, and secreted proteins) have been discovered and they have been proved to be extremely valuable tools for establishing reliable and early BC diagnosis in a minimally invasive way [146], and this potential of peripheral blood based liquid biopsy can be also fully used in the follow-up testing after anti-cancer treatment [147].The question “why this race is favoring miRNAs” is answered by their biology. The miRNAs have many properties and characteristics, such as low complexity of their molecules, tissue-specificity, stability and easy quantification and amplification, making them excellent potential biomarkers for many pathological and physiological processes. Therefore, miRNAs are becoming a point of interest in cancer detection, since they have been proven to be selectively secreted from malignant cells in the mammary gland, and are expressed differently in the blood of healthy individuals, and patients affected with BC [133]. Their onset and progress in BC detection, profiling and management thus consequently shows great promise for new options in screening, diagnosis and therapeutic interventions [148].

## 8. Tumor vs Serum miRNA Profile as a Background for miRNAs Based Screening

Cookson and al. [149] tried to answer the question of whether the circulating miRNA profiles resemble those miRNAs inside the tumor. The study analyzed plasma and tissue from patients before and after tumor resection. There were 210 miRNAs overall. Those miRNAs that have been overexpressed in plasma could be matched with those in tumors. This fact suggests that the presence of circulating miRNA can represent the solid tumor [149]. This finding has become the basis for subsequent studies focusing on BC tissue and circulating miRNAs in clinically oriented research. Many characteristics used for their detection have been identified using RNA-seq to generate profiles of miRNA expression in paired samples tumor-serum from patients with carcinoma. This resulted in a set of differently expressed miRNA between the tumor and the corresponding serum, suggesting that only a small amount of miRNA is released from primary tumor into circulation [150].

The first effort for clinical utilization of miRNAs was therefore oriented on BC screening, subsequently modified according to molecular subgroups, and later, adding the predictive potential to determine tumor biological features and aggressiveness. Initially, a wide panel of miRNAs was profiled, followed by spectra of selected miRNAs in validating studies. First of all, it was necessary to find the difference in miRNAs profiles between BC tissue and normal breast healthy tissue, e.g., miR-21, miR-125b, miR-145, mir-155 [119], followed by the finding of positive linkage between miRNAs present in the primary BC and patients plasma. Here, matching miRNAs have been subsequently validated for screening purposes, describing their expression profile or signaling function [150]. Contuinuing this approach, Heneghan et al. [148] analyzed miRNA from tumor tissues and blood samples by qRT-PCR. They quantified the level of 7 candidate miRNA of 148 patients with BC and 44 health controls. Overexpressed levels of miR-195 and let-7a reflected the presence of tumors and when these 2 miRNAs were evaluated two weeks after surgery, their expression was very low [148]. Others have conducted similar studies analyzing circulating miRNAs in patients with BC compared to healthy controls. They concluded that a plasmatic level of miRNAs could be a valid distinguishing biomarker for BC, finding a significantly higher level of several miRNAs for this purpose in their studies [151].

Nowadays, circulating miRNAs associated with neoplasia have the potential to detect cancer even in its earliest stages. It was proved that miRNAs can be used as screening tool for BC (e.g., panel of miR-127-3p, miR-148b, miR-376a, miR-376c, miR-409-3p, miR-652 and miR-801), having high distinguish ability between healthy women, and those with benign, as well as malign breast tumors, especially with better discriminatory power in younger women [148,152]. Moreover, circulating miRNAs are also to differentiate between various tumors, e.g., in compliance with their histological features such as hormone receptors or the state of lymph nodes in BC patients (expression of miR-10b, miR-373, miR299-5p, miR-411, miR-215 and miR-452 in nodal positive patients) [153], showing high specificity and sensitivity indicating metastatic disease [154]. Furthermore, a panel including miR-200a, miR-200b, miR-200c, miR-210, miR-215 and miR-486-5p can predict the onset of metastasis for up to 2 years prior to clinical diagnosis in BC patients [155]. 

The extremely valuable predicting role of circulating miRNAs showed that some of them, e.g., overexpression of miR-302b and miR-425, can be associated with early BC stage [151], or miR-182 [156], miR-155 [157], and miR-21 [158]. In particular, miR-155 and multifunctional miR-21 have recently been of high scientific interest. Meta-analyses based on relevant articles collected from several scientific databases showed that high levels of their expression correlate with detection of early stages of the disease in screening approach, and also with creating distant metastases [159,160,161]. For interest, meta-analysis involving 3 studies with 184 patients showed a screening biomarker diagnostic value with sensitivity of 79% (95%CI: 72–84%) and a specificity of 85% (95%CI: 75–92%) for mi-R155 [162]. In addition, plasmatic miR-21 and miR-155 correlate with tumor receptor status.

Another important feature of miRNAs presented in extracellular liquids is fact that they can play a role in cross-talk of cancer cells with cells of surrounding tissue, potentiating their use as biomarkers in BC [163,164]. From the known circulating miRNAs and miRNAs expressed in tissues, the evidence of abnormal activation in BC patients extends to the circulating miR-16, miR-18a, miR-21, miR-145, let-151a, miR-155 and the tissue-specific miR-7, miR-21, miR-145, miR-155/154, miR-182, miR-203, miR-213 suggesting their values as non-invasive marker, and in addition as a potential approach to overcome chemo-resistance [165].

Others correlate with treatment response and may have significant utility as predictive markers or may serve as non-invasive predictors for tumor relapse and overall survival, e.g., in triple-negative BC patients (miR-18b, miR-103, miR-107 and miR-652). Furthermore, this 4-miRNAs signature is capable of distinguishing tumors from patients with early relapse to those without recurrence [166]. This therefore makes this blood-based signature a potential risk predictor for distinguishing metastatic disease from recurrence in the early disease stage, serving as blood-based screening tool for BC relapse. This concept is nor predictive in general, nor cancer type specific. E.g. high levels of serum miR-19a may represent a biomarker for favorable clinical outcome in patients with metastatic HER2-positive BC [167].

Singling out the current knowledge in these molecules, provides blood-based miRNA liquid biopsy also the most valuable opportunity to forgo invasive methods such as tissue biopsy and associated complications in BC diagnosis, and the screening of relapse in clinical praxis [168]. Circulating miRNAs associated with BC screening approach are summarized in Table 2.

## 9. The Role of Circulating miRNAs in Profiling of BC at the Time of Sampling for Screening

As positivity of hormone receptors and estrogen signaling pathway play an important role in cancer development, progression and therapeutic response, neither tissue specific nor circulatory miRNAs reflect the endocrine tumor status. It was proved that miRNA serum profile is dependent on tumor endocrine status and may be differentially expressed (e.g., miR-21, miR-155) in the serum of women with hormone sensitive compared to women with hormone insensitive BC. Its serum concentration was found to be lower in PR tumor positivity [190]. Contrary to that, concentration of miR-182 levels was significantly increased in patients with PR + BC. Considering this fact, miR-155 and miR-182 were suggested as valued plasma biomarker for Luminal type BC diagnosis [156]. A validating study providing deeper insight into the underlying molecular portrait of Luminal A-like BC subtype selected from initial 76 deregulated miRNAs for further analysis 10 miRNAs (miR-19b, miR-29a, miR-93, miR-181a, miR-182, miR-223, miR-301a, miR-423-5p, miR-486-5 and miR-652). The biomarker potential was confirmed by RQ-PCR for four miRNAs (miR-29a, miR-181a, miR-223, and miR-652) and by binary logistic regression for three miRNAs (miR-29a, miR-181, and miR-652). A combination of these three miRNAs could reliably differentiate between cancers and controls. The expression profiles of these three miRNAs in plasma in combination with mammography, could have potential to facilitate accurate subtype-specific BC detection [191]. As with the luminal type, plasmatic miRNAs (e.g., miR-130a, miR-146a, miR-373) also differed between HER2-positive and -negative tumors [192,193,194], and the miRNAs distinguishing potential for plasma-based screening was also showed for TNBC. Mishra et al. [195] proclaimed miR-195-5p and miR-495 as prospective circulating surrogate molecular markers for early detection of either Luminal or TNBC [195]. In addition, the other circulating miRNAs (miR-16, miR-21 and miR-199a-5p) have proved this concept, finding them to be underexpressed when compared with non-TNBC. Moreover, plasma miR-199a-5p expression in TNBC significantly differed in pre and postoperative levels, and the expression levels were associated with disease stage. These results suggest that the miR-199a-5p is a TNBC-specific marker with diagnostic value and strong insight into targeted therapy during the treatment of TNBC [196]. All above mentioned just confirmed the strong leading role of miRNAs in non-invasive approach for BC screening and management. Moreover, Zhu et al. [190] highlighted that the stability of miRNAs as such screening biomarkers, by examining differential expression in the samples of patients, is safe even for samples have been conserved for 10 years [190], and if Zang et al. [197] showed the sensitivity and specificity for BC diagnosis for miRNA-30a at 74.0 and 65.6%, respectively, overweighting the sensitivities of conventional circulating tumor markers CEA and CA153 being 12.0 and 14.0%, respectively [197]. miRNAs based screening and prediction of BC is very valuable for clinical management and also in patients with a genetically increased risk for disease development. To identify a prognostic marker among asymptomatic women without a BC diagnosis, however with high risk predictive factors for developing the tumor, was an aim of a study by Taslim et al. [198], who performed genome analysis of 41-miRNA model expression in breast tissue in women without tumor with high and low BC risk (based upon Gail risk model), they have revealed miRNAs correlating with high risk for BC developing. Moreover, it was reported that altered or disrupted serum concentration of selected miRNAs, led to development of BC among these women within next 18 months [198]. All these results serve as proof-of-principle that miRNAs in women without BC may be useful for predicting BC risk and/or as an adjunct biomarker for BC early detection in screening programs among those who already developed cancer. The miRNAs identified herein may be involved in breast carcinogenic pathways because they were first identified in the breast tissues of healthy women. Circulating serum miRNAs predicting BC profile (ER, PR, TNBC, Her2+ status, stage, nodal affection) are shown in Table 3.

## 10. Pros and Cons for miRNAs in BC Screening and Management

In the context of personalized medicine, the achievement of CTC cultures and cell free nucleic acids via the liquid biopsy provides outstanding potential to noninvasively diagnostics, prediction, and monitoring of the changing patterns of drug susceptibility in individual patients as their cancer cells acquire new mutations [104,200] Analysis of cfDNA in BC individuals provides an opportunity for non-invasive sampling of tumor DNA and supports its clinical validity as a promising ‘liquid biopsy’ tumor biomarker [201]. Despite that the quantification of miRNAs and cfDNAs within BC screening in dual use mammography does not exist in literature, the clinical diagnostic may serve as a useful tool in BC diagnosis. Specific attention is placed on short miRNAs, therefore miRNA profiling in individuals may be a promising biomarker and prediction tool that could be utilized in all phases of carcinogenesis within personalized management of breast carcinoma [6,208]. However, miRNA detection as a part of liquid biopsy biomarkers still needs to be validated. There are various limitations of circulating microRNAs as biomarkers of BC. Up to now, a number of studies have been focusing on selected miRNAs that could be applicated as prognostic or predictive biomarkers. Interestingly, certain levels of potential miRNAs occur in healthy subjects as well as in patients’ blood or plasma samples. Therefore, alterations of miRNAs expression levels between controls and patients are generally quite low [207]. Importantly, the origin of miRNAs as a part liquid biopsy can influence the effectiveness of this non-invasive diagnostic method. It is well-known that the majority of miRNAs in blood are packed in extracellular vesicles such as macrovesicles or exosomes [209]. Numerous studies analyzed different miRNA expression profiles in plasma, serum and peripheral blood exosomes in BC patients in comparison with healthy individuals [207,210]. These findings suggest the fundamental importance of selecting proper sampling methods for the quantification of circulating miRNAs. Furthermore, single miRNA as a diagnostic and prognostic biomarker has limitations in attributes, including specificity and sensitivity. Moreover, levels of individual miRNA could be overlapped between patients and healthy controls and lead to generation of false positive or false negative results [211]. Nowadays, it is an active field of cancer research because it is necessary to clarify its biological context in body fluids. Moreover, even though several miRNAs have been identified as biomarkers solely or in signatures in multiple studies, many reports differ in their opinion on the detected miRNA. The reason for this is most likely the variability in the design of the studies, cohort characteristics, isolation and detection methodologies or data analysis [212]. Thus, here is an urgent need for to standardize detection and quantification assays with appropriate normalization controls. Actually, for determination of circulating BC biomarkers, there are currently extensively used genomic and proteomic methods. However, they have limited multiplexing capabilities and involve multi-step, high-cost, and time-consuming processes demanding skilled people, which limits significantly their applicability for point-of-case diagnosis [146]. Therefore, there is an urgent need to develop portable, easy handling, time-efficient, cost-effective and quantitative tools for reliable determination of circulating biomarkers at different molecular levels. These analytical tools should be afterwards implemented in decentralized and resource-limited settings. Afterwards, a panel of cancer-specific circulating miRNAs should be created with corresponding tumor grades, responses to treatment, recurrence, and patient survival, which in combination with other biomarkers detectable in liquid biopsies could increase the sensitivity and specificity of cancer detection. Equally, more research studies are necessary for the establishment of feasibility of these applications between patients’ subgroups.

## 11. Conclusions and Expert Recommendations

Despite the extensive use of mammography as the gold standard for breast cancer (BC) screening, the occurrences of both – false-positive and false-negative diagnosis, as well as over diagnosis and the high expenditures, is an issue in gynecological oncology. Consequently, BC management demands new strategies to for compensate the existing deficits. New strategies should consider:

- unmet needs of young populations such as innovative screening programs for early and predictive diagnosis, for example in case of planned pregnancies to avoid pregnancy associated BC [213]
- new diagnostic tests with more predictive power for both – primary BC prevention (by risk assessment to mitigate modifiable risks) and secondary prevention to mitigate the risk of metastatic disease [26,29,214]
- the great potential of multi-level diagnostics by phenotyping and multiomics, in order to adapt the treatment algorithms to the individualized patient profiles [3,28,215,216].

Contextually, the overall concepts of predictive, preventive and personalized medicine are strongly recommended to advance the overall BC management [217]. 

Early and predictive diagnostic approaches (specifically in premenopausal women), extended and innovative screening programs focused on young female populations (with dense breast parenchyma), targeted prevention in high-risk groups, and optimized treatment concepts are necessary for better controlling of BC. A multiomics clinical approach using liquid biopsy and based on the utilization of the circulating biomarkers has a great potential to improve and enrich the cancer screening and its later management. Promising candidate biomarkers include proteins, RNA, DNA, also autoantibodies, metabolites, and lipids, which can be applied in the detection (involving the pre-invasive and early stages of the disease), diagnosis, and treatment monitoring of BC. While protein-based cancer biomarkers have been introduced in routine pathological practice for many years, nucleic acids-based biomarkers such as miRNAs are relatively new. Blood-based biomarkers for BC screening are still at the early phases of development, and many clinical/preclinical issues including cost effectiveness need to be resolved before their standard introduction into clinical practice. However, despite this, a novel approach for BC screening based on the combination of mammography with liquid biopsy based methods (miRNAs or other) is very promising for specific clinical settings, which may refine the diagnosis and lead to more personalized cancer treatment.

## Figures and Tables

**Table 1:** Categories of women and mammography screening applicability.

Categories of Women	Applicability of Mammography
Postmenopausal with fatty breasts	The breast density gradually decreases after menopause, applicable every two years
Young with very dense breast parenchyma	Low diagnostic sensitivity
Pregnancy	Mammography is not contraindicated in the first and second trimesters (sufficient shading of the uterus is necessary); USG and MRI are predominant diagnostic methods
Family history of BC	An annual mammography, supplemental imaging (MRI, USG) in dense breast tissue
Genetic predisposition to BC	An annual mammogram starting at age 25
Diagnosis of atypical hyperplasia or lobular carcinoma in situ	An annual mammogram beginning at the time of diagnosis
Average BC risk with no symptoms	An annual mammogram combined with USG in dense breast tissue

BC, breast cancer; MRI, magnetic resonance imagining; USG, ultrasonography.

**Table 2:** Circulating miRNAs associated with BC screening approach.

miRNA	Expression (BC vs. Normal)	Sample Type	References
miR-15a	Upregulated	serum	[169]
miR-18a	Upregulated	serum	[169,170,171]
miR-107	Upregulated	serum	[169]
miR-425	Upregulated	serum	[169]
miR-139-5p	Downregulated	serum	[169]
miR-143	Downregulated	serum	[169]
miR-145	Downregulated	serum	[169,172]
miR-365	Downregulated	serum	[169]
miR-155	Upregulated	serum	[157,162,172,173,174,175,176,177]
miR-1	Upregulated	serum	[178]
miR-133a	Upregulated	serum	[178,179]
miR-133b	Upregulated	serum	[178]
miR-92a	Upregulated	serum	[178]
miR-148b	Upregulated	plasma	[152,179,180]
miR-376c	Upregulated	plasma	[180]
miR-409-3p	Upregulated	plasma	[152,179,180]
miR-801	Upregulated	plasma	[180]
miR-16	Upregulated	plasma	[181,182]
miR-21	Upregulated	plasma/serum	[133,175,176,181,183,184,185]
miR-451	Upregulated	plasma	[184]
miR-145	Downregulated	plasma	[184]
miR-222	Upregulated	serum	[170,182]
miR-127	Upregulated	plasma	[152]
miR-376a	Upregulated	plasma	[152]
miR-652	Upregulated	plasma	[152]
miR-801	Upregulated	plasma	[152]
miR-484	Upregulated	serum	[186]
miR-1246	Upregulated	serum	[187]
miR-1307	Upregulated	serum	[187]
miR-6861	Upregulated	serum	[187]
miR-4634	Downregulated	serum	[187]
miR-6875	Downregulated	serum	[187]
miR-181b	Upregulated	serum	[173]
miR-24	Upregulated	serum	[173]
miR-505	Upregulated	plasma	[133]
miR-125	Upregulated	plasma	[133]
miR-96	Upregulated	plasma	[133]
miR-195	Upregulated	serum	[148]
miR-199a	Upregulated	serum	[188]
Let-7a	Upregulated	serum	[148]
miR-106a	Upregulated	serum	[175]
miR-126	Downregulated	serum	[175]
miR-335	Downregulated	serum	[175]
Let-7c	Downregulated	serum	[189]
miR-182	Upregulated	serum	[156]
miR-25	Upregulated	serum	[182]
miR-324	Upregulated	serum	[182]

**Table 3:** Circulating serum miRNAs predicting BC profile.

miRNA	Expression	ER+/ER−	PR+/PR−	HER2+/HER2−	TNBC+/−	Nodal Affection	Stage of BC	References
miR-10b	up	−	−	−	+	yes	early	[153,199,200]
miR-18a	up	−	−	−	+			[199,201]
miR-18b	up	−	−	−	+			[199,202]
miR-20a	up			+	−	yes	early	[200,203,204]
miR-21	up	−	−	+	−	yes	early/advanced	[184,200]
miR-29a	down	+	+	−	−		early	[191]
miR-34a	up	−	−	+	−	yes		[200,205]
miR-103	down	+	+	−	−	yes		[154,199]
miR-107	down	+	+		−	yes		[154,199]
miR-125a	down	−	−	+	−		early	[119,199]
miR-125b	down	−	−	+	−		early	[119,199]
miR-138	up	+				yes	early	[206]
miR-143	down	−	−	−	+		early	[196,202]
miR-153	up	−	−	−	+			[199]
miR-155	up	−	−	−	+	yes	early	[119,199,200]
miR-181a	down	+	+	−	−		early	[191]
miR-193b	up	−	−	+	−	yes		[155,205]
miR-200a	up			+	−	yes		[200,205]
miR-200b	up			+	−	yes		[200,205]
miR-200c	up			+	−	yes		[200,205]
miR-342	up	+	+	+	−		early	[109,199]
miR-373	up			+	−	yes		[193,200]
miR-375	up	−	−	+	−	yes		[154,205]
miR-429	up	−	−	+	−	yes		[155,199]
miR-484	up	+				yes	early	[206]
miR-486-5p	up	+	+	−	−	yes		[155,191]
miR-642-3p	up						early	[207]
miR-652	down	+	+	−	−	yes	early	[152,154,191]
miR-801	up	+				yes	early	[152,160,206]
miR-1202-5p	up						early	[207]
miR-1207-5p	up						early	[207]
